# Comparisons of Differential Filtering and Homography Transformation in Modal Parameter Identification from UAV Measurement

**DOI:** 10.3390/s21165664

**Published:** 2021-08-23

**Authors:** Jiqiao Zhang, Zhihua Wu, Gongfa Chen, Qiang Liang

**Affiliations:** School of Civil and Transportation Engineering, Guangdong University of Technology, Guangzhou 510006, China; zhangjq@gdut.edu.cn (J.Z.); 2111909122@mail2.gdut.edu.cn (Z.W.); 18370849122@163.com (Q.L.)

**Keywords:** unmanned aerial vehicle, digital image correlation, operational modal analysis, differential filtering, homography transformation

## Abstract

This paper proposes a differential filtering method for the identification of modal parameters of bridges from unmanned aerial vehicle (UAV) measurement. The determination of the modal parameters of bridges is a key issue in bridge damage detection. Accelerometers and fixed cameras have disadvantages of deployment difficulty. Hence, the actual displacement of a bridge may be obtained by using the digital image correlation (DIC) technology from the images collected by a UAV. As drone movement introduces false displacement into the collected images, the homography transformation is commonly used to achieve geometric correction of the images and obtain the true displacement of the bridge. The homography transformation is not always applicable as it is based on at least four static reference points on the plane of target points. The proposed differential filtering method does not request any reference points and will greatly accelerate the identification of the modal parameters. The displacement of the points of interest is tracked by the DIC technology, and the obtained time history curves are processed by differential filtering. The filtered signals are input into the modal analysis system, and the basic modal parameters of the bridge model are obtained by the operational modal analysis (OMA) method. In this paper, the power spectral density (PSD) is used to identify the natural frequencies; the mode shapes are determined by the ratio of the PSD transmissibility (PSDT). The identification results of three types of signals are compared: UAV measurement with differential filtering, UAV measurement with homography transformation, and accelerometer-based measurement. It is found that the natural frequencies recognized by these three methods are almost the same. This paper demonstrates the feasibility of UAV-differential filtering method in obtaining the bridge modal parameters; the problems and challenges in UAV measurement are also discussed.

## 1. Introduction

Longterm use of bridges inevitably causes structural damage that may lead to bridge collapse accidents. It is necessary to carry out damage detection regularly. Vibration measurement is an important step in the field of vibration-based structural damage detection [1]. The traditional vibration measurement methods, such as using acceleration sensors [2,3] and strain gauges [4], etc., have disadvantages of complicated setup and being time-consuming. Hence, some non-contact vibration measurement methods are proposed, e.g., global positioning system (GPS) [5] and laser doppler vibrometer (LDV). Though the GPS is commonly used in vibration measurement, its measurement accuracy is low for short-span bridges with high vibration frequency [6]. As the LDV system needs to be deployed on the ground underneath the bridge and supervised during the measurement, it is just suitable for short-term bridge detection [7]. With the emergence of low-price and high-resolution cameras, vision-based methods [8,9] are commonly used in vibration measurement. As a non-contact method, it provides the possibility for bridge vibration measurement at a distance, which is not available with traditional contact methods. In addition, it is more accurate and less expensive than the other non-contact methods, e.g., GPS and LDV. Among the vision-based measurement methods, the digital image correlation (DIC) method [10,11] is widely used in mechanical manufacturing, aerospace, material processing, and other fields [12]. Compared with the accelerometer method, the DIC measurement method is full-field, non-contact, and convenient. It is also used to measure the surface cracks and deformation of asphalt and concrete structures [13,14]. In recent years, the DIC method has been used to measure the displacement of the actual bridges to obtain the modal parameters and perform damage detection, which validated the effectiveness of the DIC method for field measurement [15]. However, the traditional DIC method is to place a camera in a fixed position that is less affected by the external environment, but it is not feasible for bridges that span rivers and valleys. Since the UAV is flexible and economical, it can make up for the disadvantage of traditional fixed cameras in bridge measurement.

As a new type of bridge inspection tool, the UAV equipped with high-resolution cameras can hover in a predetermined position for image collection. The image data can be analyzed to evaluate the reliability, durability, and bearing capacity of bridges [16]. Moreover, UAVs have been widely used in different industries for monitoring, such as beach problem studies [17,18], the change of coastal zone [19] topographic mapping to observe the environment [20], and constructing a map model to estimate the shoreline changes [21]. In the real measurement environment, UAVs are susceptible to wind, elevating force, component vibration, and other factors, resulting in irregular movement during their flight. The structural displacement captured in the UAV images includes both the actual displacement of the structure and the false displacement caused by the UAV movement [22]. 

### 1.1. Related Work

In order to obtain the modal parameters of bridges exactly, numerous researchers have proposed methods to eliminate the false displacements caused by the UAV ego-motion. Yoon et al. [23] used triangulation to calculate the camera parameters for each UAV video frame, and the estimated camera projection matrix can be used to recover the world coordinates of the structure and obtain the true displacement curve. Ribeiro et al. [24] used an embedded inertial measurement unit (IMU) to obtain information on the three-axis attitude angle of the UAV, through which the motion of the UAV can be calculated, so that the true displacement of the structure can be estimated. Hoskere et al. [25] proposed a high-pass filtering method to remove the low-frequency noise caused by the hovering vibration of the UAV, but this method can only be used for the case that the structural natural frequency is much higher than the noise frequency caused by the UAV motion. Yoneyama et al. [26] proposed that the planar homography transformation relationship between images before and after camera motion can be determined by at least four sets of two-dimensional points. According to this algorithm, Chen et al. [27] used the homography transformation to eliminate the false displacements generated by the UAV motion and achieved good results. However, this method can only be applied to the case that the reference points and the target points are on the same plane, that is, the fixed correction frame must be coplanar with the bridge plane. Therefore, Wu et al. [28] used Zhang’s calibration method to calculate the projection matrix (intrinsic and extrinsic matrices) of each frame of UAV images, which can be used to recover the 3D world coordinates of the structure and obtain its real displacement curve. This method effectively overcomes the restrictive condition that the reference points and the target points can only be located in the same plane in the homography-based method, and it has been well validated in bridge model tests. Both of the above methods require a fixed correction frame as the reference, which is difficult to find or arrange in the actual bridge measurement. 

### 1.2. Contribution Summary of the Paper

Since UAV movement is a random process, it is expected that the differential filtering method can eliminate this random component in the measured signals to get the correct modal parameters. Differential filtering as a signal processing method is often used for signal enhancement and recovery [29]. The signal obtained by the differential filtering is input into the acquisition instrument for corresponding modal analysis to obtain the modal parameters of the structure. Operational modal analysis (OMA) method [30] is a technique for extracting modal parameters from vibration response signals [31]. Compared with traditional experimental modal analysis (EMA) methods, the OMA only needs to collect the dynamic responses of the structure without using the excitations of the structure [32]. The OMA usually uses the power spectrum density (PSD) to identify the natural frequencies of the structure [33] and the ratio of the PSD transmissibility (PSDT) [34] to determine the mode shapes of the structure [35].

This paper proposes a differential filtering method to remove the random UAV movement from the measured signals, through which the modal parameters of bridges can be obtained directly. A UAV is used to collect images of bridge vibrations, and then the DIC technology is used to track the points of interest to obtain their displacement time-history curves that contain the random UAV movement. The displacement time-history signals are processed by the differential filtering technology; the processed time-history signals are imported into the modal analysis system; the modal parameters are extracted by the OMA method. Finally, the feasibility of the differential filtering method in signal processing is verified by comparing with the homography-based and the accelerometer-based methods.

## 2. Methods

The first step of this work is to process the UAV video frames by the DIC method; the second and third steps are to process the displacement data by using the homography-based method and differential filtering method, separately. Finally, the OMA method is used to estimate the modal parameters.

### 2.1. DIC Method

**Figure 1:**
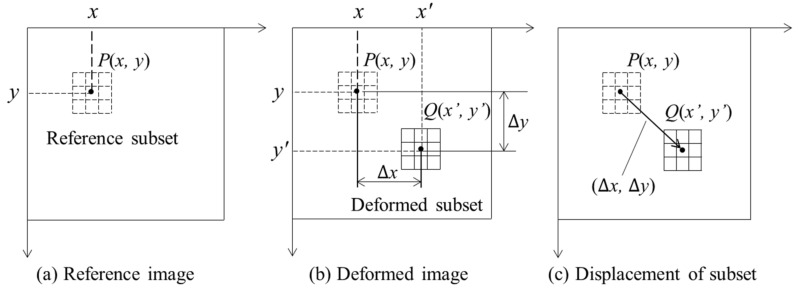
Tracking point movement by DIC method.

The tracking principle of the DIC is shown in Figure 1. A point of interest P(x,y) surrounded by the reference sub-region is the tracked point. The displacement between the reference sub-region and the deformed sub-region is (Δx,Δy), and the coordinate value of the center point $Q(x^{\prime },y^{\prime })$ of the deformation sub-region is $x^{\prime }=x+\Delta x$ and $y^{\prime }=y+\Delta y$. The correlation between the reference sub-region and the deformed sub-region can be expressed as [36,37]:

(1) $$C(\Delta x,\Delta y)=\frac{\iint_{S}I(x,y)J(x+\Delta x,y+\Delta y)dxdy}{\sqrt{\iint_{S}I^{2}(x,y)dxdy}\cdot \sqrt{\iint_{S}J^{2}(x+\Delta x,y+\Delta y)dxdy}}$$

The correlation coefficient varies with the displacement (Δx,Δy). The actual displacement of P will maximize the correlation, C(Δx,Δy); i.e., the deformed sub-region is most similar to the reference sub-region at this displacement (Δx,Δy). The movement of the bridge model is recorded by the UAV. The recorded image frames are processed by the DIC method to obtain the time-history signals of the point of interest, and the processing time for each frame is about 2 s. Then, the time-history signals are processed by differential filtering and input into the dynamic analysis system to extract the modal parameters.

### 2.2. UAV Image Correction

**Figure 2:**
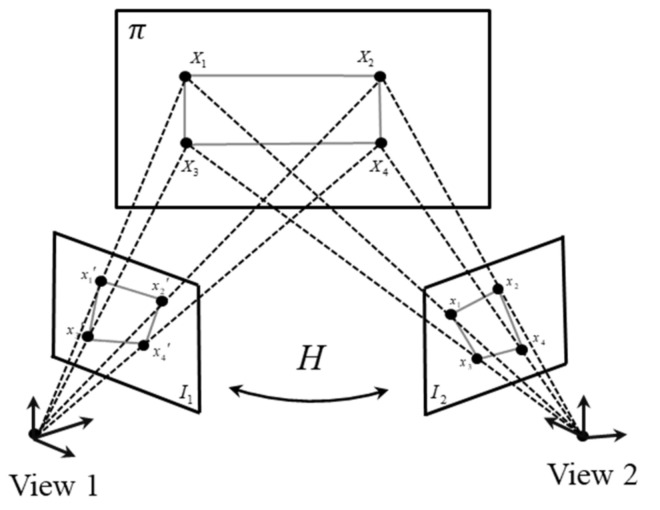
Transformation between two images.

The UAV image correction is achieved by solving the homography transformation between two different images in the space as shown in Figure 2. In this paper, the homography transformation between the two images is established by matching the positions of four static reference points that are coplanar with the target points. The homography transformation is used to correct the results of the DIC-processed UAV images; the false displacements caused by the UAV motion are eliminated, and the true displacements of the target points are obtained.

As shown in Figure 2, $I_{1}$ and $I_{2}$ are two images taken by the UAV sequentially. The first image, $I_{1}$, is used as the reference image, and the images from the second to the *n*-th are the images to be corrected. The points on $I_{1}$ and $I_{2}$ can be transformed into the same coordinate system by employing the theory of plane homography [27]:

(2) $$sp_{i}^{\prime }=Hp_{i}$$

where, H is a 3×3 homography matrix; *s* is the scale factor, which is related to the shooting distance and the actual length of the object to be measured; $p_{i}$ and $p_{i}^{\prime }$ are the pixel values of the target points in the image to be corrected and those transformed to the reference image, respectively.

The homography matrix, H, can be determined by four static reference points. Suppose that $x={\left(u,v,1\right)}^{T}$ and $x’={\left(u^{\prime },v^{\prime },1\right)}^{T}$ are the homogeneous pixel coordinates of a reference point on the original image ($I_{1}$) and deformed image ($I_{2}$), respectively. Equation (2) is expressed in homogeneous coordinates as follows:

(3) $$s\left[\begin{array}{l} u^{\prime }\\ v^{\prime }\\ 1 \end{array}\right]=\left[\begin{array}{ccc} h_{11} & h_{12} & h_{13}\\ h_{21} & h_{22} & h_{23}\\ h_{31} & h_{32} & h_{33} \end{array}\right]\left[\begin{array}{l} u\\ v\\ 1 \end{array}\right]$$

where $h_{ij}$ is the element in *i*-th row and *j*-th column of the homography matrix, H. Then the homography matrix H can be solved as:

(4) $$\left(\begin{array}{cccccccc} u_{1} & v_{1} & 1 & 0 & 0 & 0 & -u_{1}{u^{\prime }}_{1} & -v_{1}{u^{\prime }}_{1}\\ 0 & 0 & 0 & u_{1} & v_{1} & 1 & -u_{1}{v^{\prime }}_{1} & -v_{1}{v^{\prime }}_{1}\\ u_{2} & v_{2} & 1 & 0 & 0 & 0 & -u_{2}{u^{\prime }}_{2} & -v_{2}{v^{\prime }}_{2}\\ 0 & 0 & 0 & u_{2} & v_{2} & 1 & -u_{2}{v^{\prime }}_{2} & -v_{2}{v^{\prime }}_{2}\\ u_{3} & v_{3} & 1 & 0 & 0 & 0 & -u_{3}{u^{\prime }}_{3} & -v_{3}{u^{\prime }}_{3}\\ 0 & 0 & 0 & u_{3} & v_{3} & 1 & -u_{3}{v^{\prime }}_{3} & -v_{3}{v^{\prime }}_{3}\\ u_{4} & v_{4} & 1 & 0 & 0 & 0 & -u_{4}{u^{\prime }}_{4} & -v_{4}{u^{\prime }}_{4}\\ 0 & 0 & 0 & u_{4} & v_{4} & 1 & -u_{4}{v^{\prime }}_{4} & -v_{4}{v^{\prime }}_{4} \end{array}\right)\left(\begin{array}{l} h_{11}\\ h_{12}\\ h_{13}\\ h_{21}\\ h_{22}\\ h_{23}\\ h_{31}\\ h_{32} \end{array}\right)=\left(\begin{array}{l} {u^{\prime }}_{1}\\ {v^{\prime }}_{1}\\ {u^{\prime }}_{2}\\ {v^{\prime }}_{2}\\ {u^{\prime }}_{3}\\ {v^{\prime }}_{3}\\ {u^{\prime }}_{4}\\ {v^{\prime }}_{4} \end{array}\right)$$

If there are more than four reference points, the least square method is used to solve the homography matrix.

Suppose that the target point on the *i*-th (i=1,2,3…n) image is $q_{i}(u_{i},v_{i})$. The real coordinate $q_{i}^{\prime }$ of the target point after correction can be obtained as follows:

(5) $$q_{i}^{\prime }=\frac{1}{s}Hq_{i}$$

where, $s=h_{31}u_{i}+h_{32}v_{i}+1$. This formula realizes the correction of the target point and obtains the true position of the target point. In accordance with the above method, the second to *n*-th images can be projected into the coordinate plane of the original image in turn to solve the true displacement of the structure. The real displacement of the target point is $q_{i}^{\prime }-q_{1}$.

The unit pixel length can be obtained through the calibration of pixels, η=l/α, where l is the actual length between two reference points; α is the number of pixels between them. The pixel size and the corresponding actual distance between two reference points can be converted by using the conversion coefficient η.

### 2.3. Differential Filtering

Differential filtering is used to solve the problems of zero drift and signal noise in UAV measurement signals. The acceleration signal can be extracted from the displacement time-history by differential filtering:

(6) $$y_{k}=x_{k+2}+x_{k}-2x_{k+1}$$

where $x_{k}$ is the original signal; $y_{k}$ is the new signal after the second-order differential filtering [38].

In the bridge response signal, the energy contained in the high-order mode shapes is relatively low. In the PSD curve, the amplitude at the first-order natural frequency may be much larger than that of the other ones, which may cause difficulty in identifying the high-order natural frequencies. For a sinusoid excitation, $f=F_{0}\sin(\omega t)$, the displacement response of the system is $x=X_{m}\sin(\omega t+\varphi )$ with the amplitude:

(7) $$X_{m}=\frac{F_{0}/m_{r}}{\sqrt{{\left(\frac{k_{r}}{m_{r}}-\omega^{2}\right)}^{2}+{\left(\frac{c_{r}}{m_{r}}\omega \right)}^{2}}}$$

where $m_{r}$, $c_{r}$, and $k_{r}$ are the *r*-th order modal mass, damping, and stiffness of the system, respectively. The results of the displacement signal and its maximum amplitude-frequency response after first-order and second-order derivation are shown in Table 1 (where, $\Omega_{r}=\sqrt{\frac{k_{r}}{m_{r}}}$). Because the damping ratio of the bridge structure is small, the peak frequencies of the PSD signal curve are close to the structural natural frequencies. Applying the differential filtering twice will increase the amplitude $\omega^{2}$ times and amplify the high-frequency part of the signal, which is conducive to the identification of the natural frequencies to a certain extent.

### 2.4. Operational Modal Analysis

The response transmissibility (RT) is usually used in the operational modal identification of the structure. In a multiple-degree-of-freedom system, the response transmissibility is defined as $T_{io}(\omega )$ [39]:

(8) $$T_{io}(\omega )=\frac{X_{i}(\omega )}{X_{o}(\omega )}$$

where $X_{i}(\omega )$ and $X_{o}(\omega )$ are the Fourier transforms of the corresponding responses $x_{i}(t)$ and $x_{o}(t)$ at degrees of freedom i and o, respectively.

The PSD transmissibility is expressed as:

(9) $${\hat{T}}_{io}(\omega )=\frac{S_{i,o}(\omega )}{S_{o,o}(\omega )}=\frac{X_{i}(\omega )X_{o}^{*}(\omega )}{X_{o}(\omega )X_{o}^{*}(\omega )}$$

where, $S_{i,o}(\omega )$ is the cross power spectrum density (CPSD) of the responses $x_{i}(t)$ and $x_{o}(t)$; $S_{o,o}(\omega )$ is the PSD of the response $x_{o}(t)$, while $X_{o}^{*}(\omega )$ is the conjugate complex number of $X_{o}(\omega )$. As proved in Devriendt and Guillaume [40], the PSD transmissibility is equivalent to:

(10) $${\hat{T}}_{io}(\omega )=\frac{X_{i}(\omega )}{X_{o}(\omega )}=\frac{H_{ip}(\omega )\cdot F_{p}(\omega )}{H_{op}(\omega )\cdot F_{p}(\omega )}=\frac{H_{ip}(\omega )}{H_{op}(\omega )}=T_{io}(\omega )$$

where $H_{ip}(\omega )$ is the frequency response function (FRF) [35]. $F_{p}\left(\omega \right)$ is the Fourier transform of $f_{p}\left(t\right)$. The above formula shows that the PSD transmissibility is equal to the ratio of the FRF measured at points *i* and *o* measured when the excitation force is applied at point *p*. From Equation (10), we have:

(11) $$\underset{\omega \to \omega_{r}}{\lim}{\hat{T}}_{io}(\omega )=\frac{H_{ip}(\omega )}{H_{op}(\omega )}=\frac{\varphi_{ri}}{\varphi_{ro}}$$

${\hat{T}}_{io}(\omega )$ is expressed as the ratio of the *r*-th mode shape values at points i and o, which is independent of the magnitude of the exciting force. With the same *o*-th degree of freedom as a reference and the *i*-th degree of freedom being changed $i\;\left(i.e.,i=1,2,\ldots ,N\right)$, the *r*-th modal shape can be obtained as follows:

(12) $$\left[\begin{array}{l} \varphi_{r1}\\ \varphi_{r2}\\ \;\vdots \\ \varphi_{rN} \end{array}\right]=\varphi_{ro}\left[\begin{array}{c} {\overset{⌢}{T}}_{1o}(\omega_{r})\\ {\overset{⌢}{T}}_{2o}(\omega_{r})\\ \vdots \\ {\overset{⌢}{T}}_{No}(\omega_{r}) \end{array}\right]$$

From Equation (12), the modal shape of the structure can be obtained as follows:

(13) $$\varphi_{r}=\underset{\omega \to \omega_{r}}{\lim}\left\{{\overset{⌢}{T}}_{1o}(\omega ),{\overset{⌢}{T}}_{2o}(\omega ),\ldots ,{\overset{⌢}{T}}_{no}(\omega )\right\}$$

Substituting Equation (9) into Equation (13):

(14) $$\varphi_{r}=\underset{\omega \to \omega_{r}}{\lim}\left\{S_{1,o}(\omega ),S_{2,o}(\omega ),\ldots ,S_{n,o}(\omega )\right\}$$

**Figure 3:**
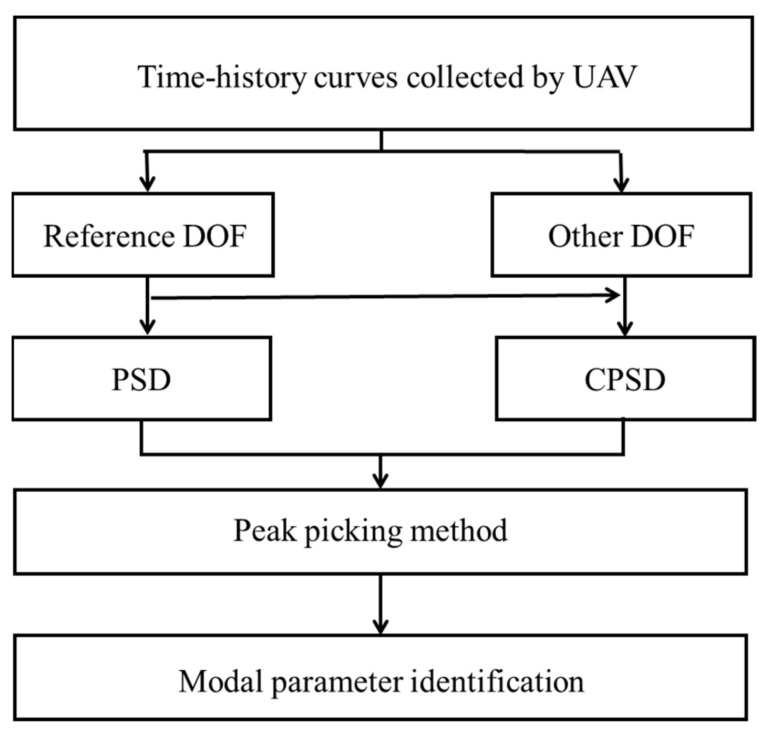
Flowsheet of operational modal analysis.

The process of using OMA to identify structural modal parameters is shown in Figure 3. Firstly, the natural frequency of the structure can be identified by analysing the PSD curve [41]. Then, the PSD transmissibility is used to calculate the mode shape.

## 3. Experiment

The experiment is conducted in an outdoor environment. The experimental setup, instrument, plan, and goal are introduced in the following.

### 3.1. Experimental Setup and Instrument

**Figure 4:**
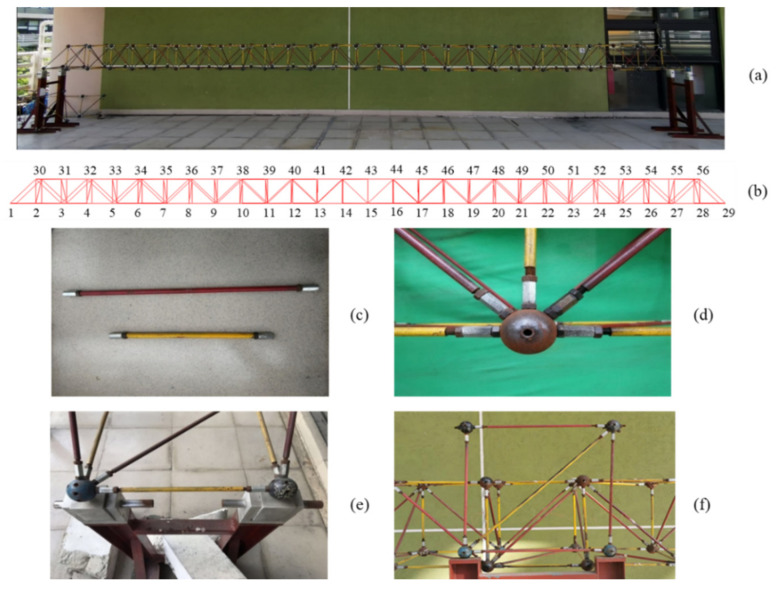
Experimental model: (**a**) Overall model; (**b**) Model node number; (**c**) Rods; (**d**) Component connection; (**e**) Supports; (**f**) Rectangular correction frame.

The experimental model (Figure 4) is a 28-span steel frame with a length of 9.80 m. Each span has dimensions of 0.35 m × 0.35 m × 0.35 m. The model consists of rods, bolted balls, bolts, and nuts. The length of the yellow rods and red rods is 0.35 m and 0.5 m, respectively, and the bolted balls have a diameter of 0.05 m. Both ends of the model are hinged.

In order to improve the image resolution and tracking accuracy of the DIC method, the UAV shooting distance is shortened in this experiment. The UAV is used to take pictures at a distance of 3 m and records one half of the model.

**Figure 5:**
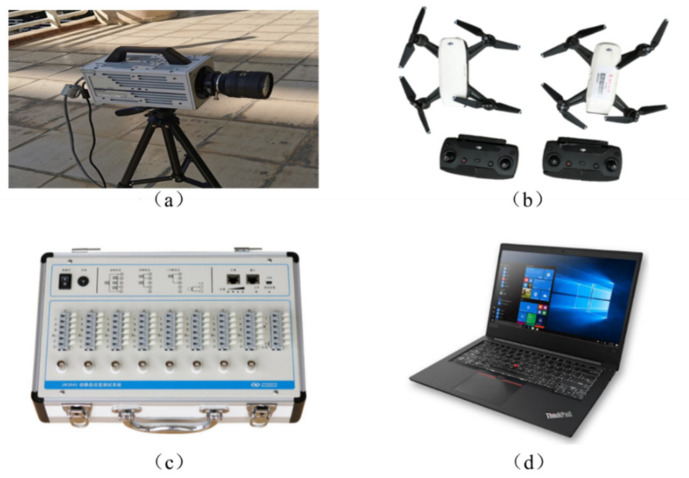
Experimental equipment: (**a**) Fixed camera; (**b**) DJI drone; (**c**) JMTEST acquisition instrument; (**d**) JMTEST dynamic acquisition software.

The experimental equipment for modal analysis (Figure 5) includes the following:

1. Digital camera (FASTCAM SA3, Photron Inc., Tokyo, Japan) for recording model vibration;
2. DJI’s quadrotor drones (Spark, Da-Jiang Innovations, Shenzhen, China) with a high-resolution camera with a sampling frequency of 30 frames per second and a resolution of 1920 × 1080 pixels;
3. A signal acquisition system for collecting signals (JM3840, Jing-Ming Technology Inc., Yangzhou, China);
4. A laptop computer connected to the acquisition system.

The displacement response is collected and processed by the dynamic acquisition system (JM3841), and then the structural modal parameters (frequencies and mode shapes) are obtained from the processed PSD and CPSD curves.

### 3.2. Experiment Plan and Goal

**Figure 6:**
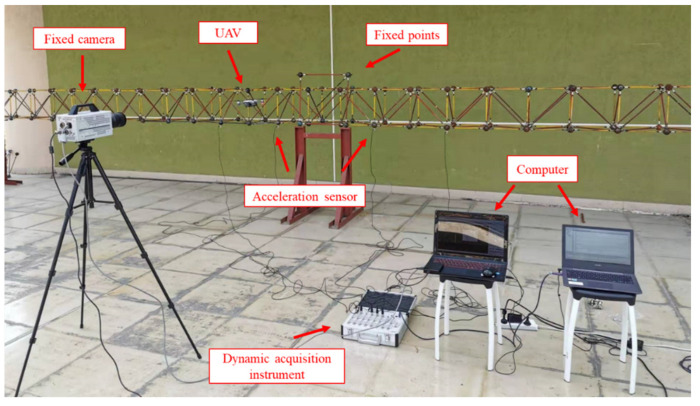
Experimental layout.

The experiments are designed to verify the effectiveness of the proposed differential filtering method in obtaining the modal parameters from UAV measurements. The experimental layout is shown in Figure 6. In order to simulate the actual measurement environment, all experiments are performed outdoors. The displacement signals of the experimental model are collected by the drone, and the measurements of the fixed camera are used as the reference. In order to study the feasibility of differential filtering in processing UAV signals, a series of measurements are carried out for different data processing methods.

As shown in Table 2, the recording times for the drone, fixed camera, and acceleration sensor are 60 s. The fixed camera has a sampling frequency of 2000 frames per second and a resolution of 1024 × 1024 pixels. It will record 120,000 images for processing. The drone has a sampling frequency of 30 frames per second and a resolution of 1920 × 1080 pixels. It will record 1800 images for processing. The sampling frequency of the acceleration sensor is set to 50 Hz/s. The processing time for each frame is about 2 s when performing the homography transformation.

The main goals of this experiment include the following:

(a) Verifying the correction accuracy of the UAV results by comparing them with fixed cameras. The image sequence of the UAV is corrected by homography transformation to obtain the true displacement time-history signal, which is imported into the dynamic acquisition software with the time-history signal of the fixed camera to obtain the modal parameters. The results are compared with those of the fixed camera to demonstrate the feasibility of UAVs in actual vibration measurement.
(b) Verifying the accuracy of the differential filtering method by comparing with homography-based correction results. The uncorrected time-history signals of UAV measurements are processed by the proposed differential filtering, and the processed results are input into the dynamic acquisition software to obtain the modal parameters, which are compared with those identified from the other two methods: accelerometer measurements and homography-based correction of UAV images.

## 4. Results

The experimental results are presented in three parts. The first part compares the measurement results of the UAV and the fixed camera. The second part describes the different processing effects between the differential filtering and the homography correction. The last part compares the modal parameters extracted by three methods: differential filtering, homography correction, and accelerometer measurement.

### 4.1. Measurement Results of UAV and Fixed Camera

The largest vibration amplitude of the bridge model is at the middle of the span, thus Node 15 is selected as the target point for comparative study.

**Figure 7:**
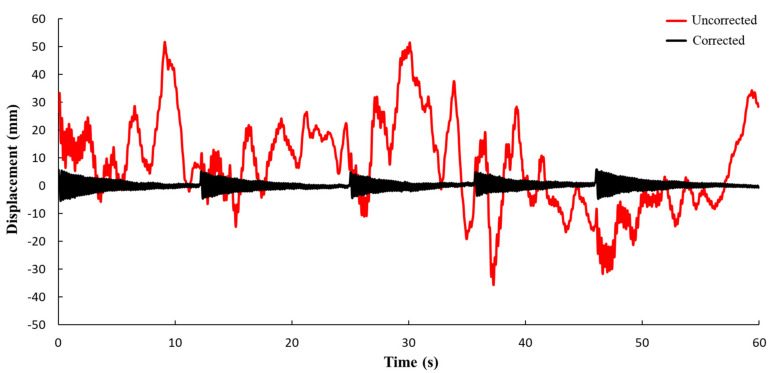
Comparison of displacement time history curves (corrected and uncorrected).

The displacement (corrected and uncorrected) of the target point is shown in Figure 7. During the experiment, the frame is excited five times, hence there are five peaks on the corrected curve. The uncorrected displacement includes not only the real displacement of the structure, but also the false displacement caused by UAV motion. After the image correction, the displacement curve shows the characteristics of free vibration.

**Figure 8:**
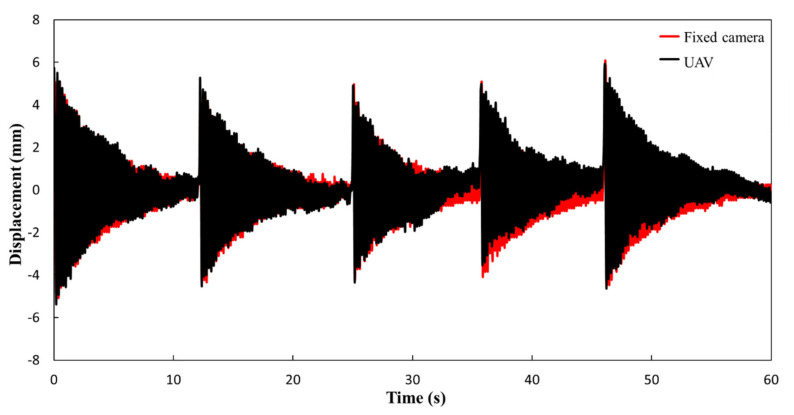
Comparison of recorded signal from fixed camera and UAV (corrected).

As shown in Figure 8, the false displacement can be eliminated by geometric correction, and the real displacement of the target point can be obtained.

### 4.2. Comparisons of the Processing Effects of Differential Filtering and Homography Transformation

**Figure 9:**
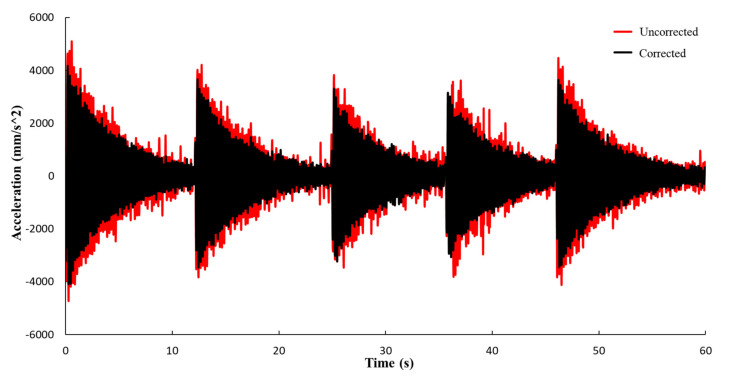
2nd-differential curves of two signals (uncorrected and corrected).

The time-history curve of the target point is processed with second-order differential filtering and homography transformation, and the results are shown in Figure 9. After differential filtering, the UAV displacement time curve is transformed into an acceleration-time curve.

The results of homography transformation and differential filtering show the same free vibration attenuation characteristics by comparing the two curves in Figure 9. The curve of the differential filtering of corrected UAV signals is smoother than that of direct differential filtering of original UAV signals. Based on the above results, the feasibility of using differential filtering without geometric correction can be preliminarily determined.

### 4.3. Modal Parameters Identified from Differential Filtering, Homography Transformation and Accelerometer Measurements

**Figure 10:**
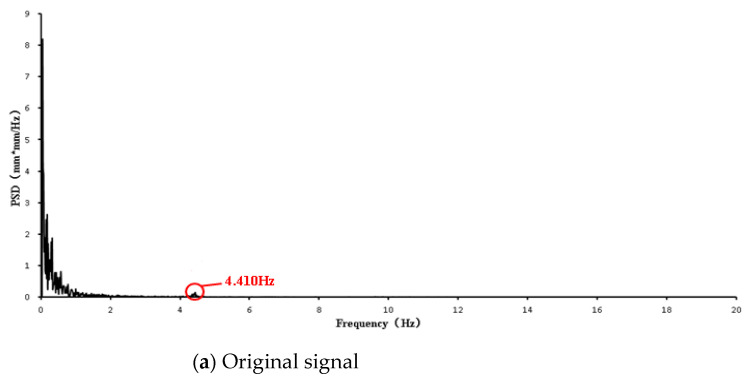

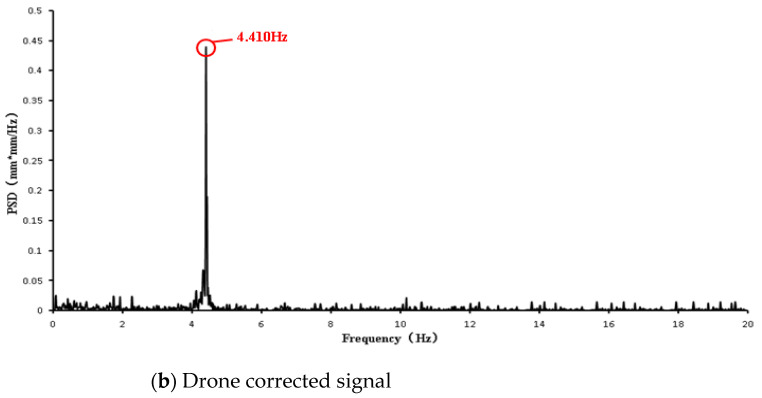
PSD function curves of two signals: Original and corrected.

The PSD curves of the original signal and the corrected signal of UAV measurements are shown in Figure 10a,b.

Figure 10a showed zero drift and signal noise. It is difficult to pick up the natural frequency of the structure from the PSD curve of the original UAV signal (Figure 10a), while the PSD curve of the corrected UAV signals shows a frequency at 4.410 Hz (Figure 10b). Figure 10b showed that the UAV correction is able to solve the problems of zero drift and signal noise in UAV signals.

**Figure 11:**
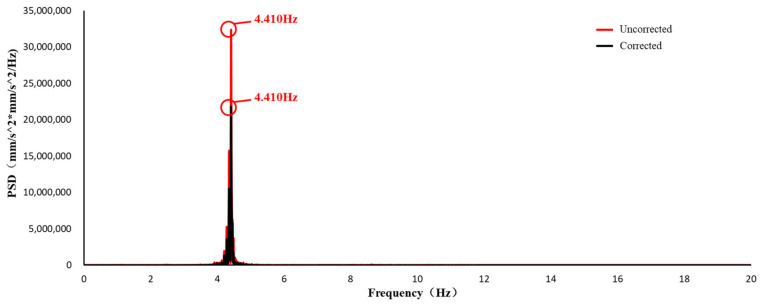
Modal parameter identification results of differential filtering of UAV signals.

**Figure 12:**
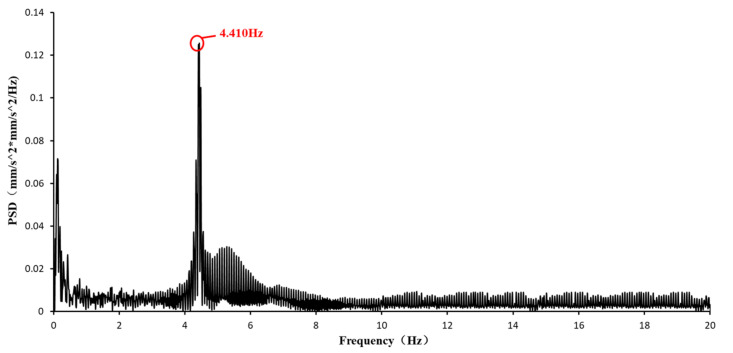
Modal parameter identification results of accelerometer measurement.

The original signal and corrected signal of UAV measurement are processed by the second-order differential filtering; the modal parameters are shown in Figure 11. As illustrated in Figure 11, the differential filtering can also solve the problems of zero drift and signal noise in UAV signals as same as the UAV correction. For comparison, the modal parameters identified with the accelerometer signal are shown in Figure 12. Table 3 shows that the first-order natural frequency identified by the three methods is 4.410 Hz.

**Figure 13:**
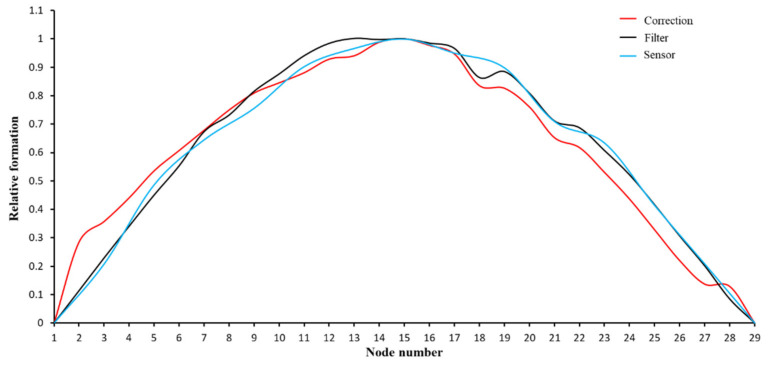
Comparison of the mode shapes identified by three processing methods.

In addition, the first-order mode shapes obtained by differential filtering, geometric correction, and accelerometer methods are shown in Figure 13. The fitting degree between the mode shape is commonly judged by modal assurance criterion (MAC) [42]. With the first-order mode shape identified by the accelerometers being the reference, the MAC values between it and the mode shape identified from the corrected UAV signal after differential filtering is 0.995, while the MAC between it and the mode shape identified from the original signal after differential filtering is 0.997. Comparison of the processed results demonstrated that the differential filtering can replace the geometric correction for modal parameter identification.

## 5. Discussion and Conclusions

In this paper, the UAV images processed by the DIC and the obtained displacement signals are either corrected by the homography transformation or the differential filtering method. The OMA is carried out to identify the corresponding modal parameters, and the feasibility of the differential filtering replacing the UAV correction in identifying modal parameters is proved.

Section 4.1 and Section 4.2 show that data acquisition for structures using UAVs based on homography transformation has good results. In addition, there are some differences among the results obtained by the two methods (UAV signals processed by the differential filtering and geometric correction). The main reason is that the precision of the DIC tracking algorithm is of limitation.

Section 4.3 demonstrated that the modal parameters identified by the differential filtering and geometric correction are 4.410 Hz, which is consistent with the results from the accelerometer measurements. After differential filtering, the effect of zero drift on the PSD curves is suppressed. The homography-based method is implemented by geometric correction first, and then the modal parameters are extracted from the corrected displacements. The differential filtering method proposed in this paper can directly extract the modal parameters from the uncorrected displacement. In practical engineering measurement, it is important to extract the modal parameters of bridges easily and quickly. In order to find the most suitable method for obtaining modal parameters, it is necessary to compare the two methods in terms of time and convenience. The comparisons of the modal parameters identified by the two processing methods proved that differential filtering is feasible to replace geometric correction in UAV data processing. This will greatly shorten the modal identification time, and it avoids the manual arrangement of the fixed correction frame, which can facilitate measurement. The differential filtering method, however, has the feature of high-pass filtering that can change the nature of the original discrete signal and may introduce other noises. How to avoid these effects is the focus of follow-up research.

The following conclusions can be drawn from the above work:

(1) Under the same experimental conditions, UAVs can replace fixed cameras to accomplish data acquisition. The real-time history signals can be obtained after the homography transformation. Compared with accelerometer measurement, the DIC method is non-contact and full-field. More target points can be selected for measurement to improve the identification accuracy of mode shapes. Combination of the DIC technology with UAV measurement can greatly improve the efficiency of modal identification of real bridges.
(2) Differential filtering is used to remove the zero drift and signal noise in UAV signals. Differential filtering can replace geometric correction for data processing and the modal parameters can be directly identified without obtaining the real structural displacement. Hence, differential filtering can greatly simplify modal identification from UAV measurement.

In conclusion, the differential filtering method proposed in this paper is simple to operate and applicable to modal identification in bridge engineering. Compared with the homography-based method, the proposed method does not require either fixed reference points or image geometric correction. By directly differentiating the collected bridge vibration data twice, the zero-point drift of the PSD curve caused by the drone motion is filtered out, and the high-frequency part of the signal is also amplified, which is beneficial to the identification of higher-order vibration frequencies. Eventually, the natural frequencies and mode shapes can be obtained directly from the uncorrected displacement data that contains UAV motion. This greatly improves the efficiency of bridge vibration measurement using UAVs. With the further development of UAV technology and image processing algorithms, vibration measurement technology based on UAVs will play a more important role in dynamic analysis and damage identification of bridges.

## Figures and Tables

**Table 1:** Differential influence on signal.

Index	Peak Frequency	Extreme Value
Displacement	$\Omega_{r}\sqrt{1-2\zeta^{2}}$	$X_{m}=\frac{F_{0}/m_{r}}{\sqrt{{\left(\frac{k_{r}}{m_{r}}-\omega^{2}\right)}^{2}+{\left(\frac{c_{r}}{m_{r}}\omega \right)}^{2}}}$
First-order derivative	$\Omega_{r}$	$X_{m}=\frac{\omega F_{0}/m_{r}}{\sqrt{{\left(\frac{k_{r}}{m_{r}}-\omega^{2}\right)}^{2}+{\left(\frac{c_{r}}{m_{r}}\omega \right)}^{2}}}$
Second-order derivative	$\Omega_{r}/\sqrt{1-2\zeta^{2}}$	$X_{m}=\frac{\omega^{2}F_{0}/m_{r}}{\sqrt{{\left(\frac{k_{r}}{m_{r}}-\omega^{2}\right)}^{2}+{\left(\frac{c_{r}}{m_{r}}\omega \right)}^{2}}}$

**Table 2:** Experimental setups of acquisition equipment.

Sampling Equipment	Sampling Frequency (Hz/s)	Resolution (Pixels)	Sampling Time (s)	Total (Frames)
Fixed camera	2000	1024 × 1024	60	120,000
Drone	30	1920 × 1080	60	1800
Accelerometer	50	/	60	/

**Table 3:** Identification results of 3 measurement methods.

Measurement Methods	Accelerometer	UAV Correction 2nd-Differential	UAV Original Signal with 2nd-Differential
First-order natural frequency	4.410 Hz	4.410 Hz	4.410 Hz

## Data Availability

Not applicable.
